# Neuropeptide Y Is an Immunomodulatory Factor: Direct and Indirect

**DOI:** 10.3389/fimmu.2020.580378

**Published:** 2020-10-06

**Authors:** Wei-can Chen, Yi-bin Liu, Wei-feng Liu, Ying-ying Zhou, He-fan He, Shu Lin

**Affiliations:** ^1^Department of Anesthesiology, The Second Affiliated Hospital, Fujian Medical University, Quanzhou, China; ^2^Centre of Neurological and Metabolic Research, The Second Affiliated Hospital, Fujian Medical University, Quanzhou, China; ^3^Diabetes and Metabolism Division, Garvan Institute of Medical Research, Sydney, NSW, Australia

**Keywords:** neuropeptide Y, immunomodulatory, immune cells, body temperature, obesity, diabetes, emotion

## Abstract

Neuropeptide Y (NPY), which is widely distributed in the nervous system, is involved in regulating a variety of biological processes, including food intake, energy metabolism, and emotional expression. However, emerging evidence points to NPY also as a critical transmitter between the nervous system and immune system, as well as a mediator produced and released by immune cells. *In vivo* and *in vitro* studies based on gene-editing techniques and specific NPY receptor agonists and antagonists have demonstrated that NPY is responsible for multifarious direct modulations on immune cells by acting on NPY receptors. Moreover, via the central or peripheral nervous system, NPY is closely connected to body temperature regulation, obesity development, glucose metabolism, and emotional expression, which are all immunomodulatory factors for the immune system. In this review, we focus on the direct role of NPY in immune cells and particularly discuss its indirect impact on the immune response.

## Introduction

Neuropeptide Y (NPY), a polypeptide consisting of 36 amino acid residues, was first isolated from the porcine brain in 1982. It belongs to the neuroendocrine peptide NPY family, which also includes peptide YY (PYY) and pancreatic polypeptide (1). The NPY is widely present throughout the body. In the central nervous system (CNS), it is distributed in the hippocampus, cerebral cortex, hypothalamus, thalamus, brain stem, and cerebellum structures (2). In the peripheral nervous system, NPY is stored in the postganglionic sympathetic nerve, and co-stored and co-released with norepinephrine (NE) (3). An increasing number of studies have found widespread existence of NPY in peripheral tissues, such as the retina, smooth muscle, intestine, bone marrow, and thymus (4–7). Moreover, NPY is also expressed in a variety of immune cells. Its extensive distribution has a series of biological effects on various processes, including food intake, energy metabolism, stem cell differentiation, blood pressure regulation, and immune regulation (2, 8–10).

**Figure 1:**
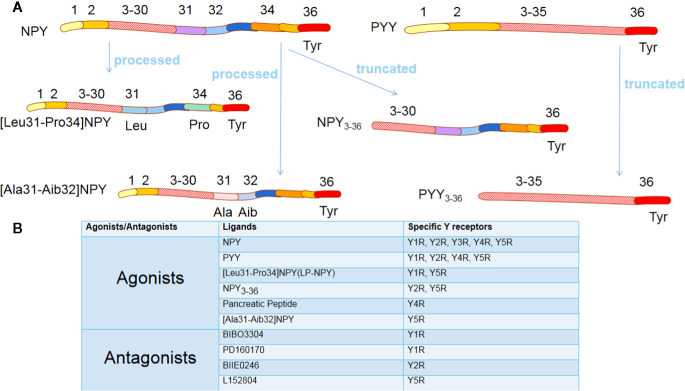
The agonists and antagonists of Y receptors. **(A)** NPY and PYY are processed into different ligands. Although these ligands have a similar amino acid composition to NPY/PYY, when the two amino acid residues of NPY/PYY are replaced or truncated, it significantly changes their affinity for different Y receptor subtypes. **(B)** The most commonly used agonists and antagonists specific for different Y receptor subtypes. NPY, neuropeptide Y; PYY, peptide YY; Tyr, Tyrosine; Leu, Leucine; Pro, Proline; Ala, Alanine; Aib, 2-aminoisobutyric acid.

NPY exerts its effects through interaction with NPY receptors (NPYRs), which are seven-transmembrane G protein-coupled receptors with different isoforms. There are six isoforms of NPY, Y1, Y2, Y3, Y4, Y5, and Y6 receptors, except Y3R, which have been cloned in mammals (11). The Y6R is inactive in primates as it loses seven transmembrane domains, but it plays a particular biological function in mice (12). Various NPYRs own corresponding specific agonists with the most robust affinities, Summarized in **Figure 1**. Though diverse, their structures are surprisingly similar, having an N-terminal which ends with tyrosine (13). Notably, dipeptidyl peptidase 4 (CD26) on the cell membrane is a serine protease that participates in the primary N-terminal truncation of NPY and PYY, leading to the formation of NPY_3-36_ and PYY_3-36_ (14) (**Figure 1A**). Promisingly, several specific NPYRs antagonists have been developed (13) (**Figure 1B**).

By utilizing specific agonists and antagonists of the NPYRs, it was shown that NPY and its receptors involved in the functional modulation of immune cells could directly act on the NPYRs of immune cells to regulate the immune response. Additionally, NPY plays a crucial role in body temperature (BT) regulation, weight control, glucose metabolism, anti-anxiety, and anti-depression, which are closely related to the immune response. Therefore, we propose an indirect effect of NPY on immune regulation. In this review, we highlight the direct role of NPY in the immune response and discuss its potential impact on the immune system.

## NPY and Its Receptors in the Immune System

### Sources of NPY in the Immune System

The NPY of the immune system is mainly derived from the sympathetic nervous system, parenchymal, and immune cells (**Table 1**). The sympathetic nerve releases NPY to act on the immune organs it innervates (15, 31, 32). Furthermore, splenic tissue sections release NE and NPY spontaneously or under electric field stimulation; subsequently, NPY acts on the Y1R to inhibit the release of interleukin-6 (IL-6) (31), suggesting that neuronal release of NPY modifies the immune response. Indeed, NPY produced by splenic sympathetic nerve endings mediates nerve communication with immune cells (15). Furthermore, NE significantly promotes the release of NPY from prostate cancer cells through the β-2 adrenergic receptors (NE-β-2AR-cAMP pathway) (18), thereby regulating the tumor immune microenvironment.

**Table 1:** Source and role of neuropeptide Y.

Sorting	Source	Role	Reference
Nonimmune tissue	Splenic sympathetic nerve	Mediates the communication between nerve and Tyrosine Hydroxylase+ leukocyte	(15)
	Retina	Regulates immune cells involved in maintaining the immune immunity of the eye	(4, 16, 17)
	Myc-CaP cells	Promotes the migration of macrophages and the secretion of IL-6 to participate in the regulation of the tumor microenvironment	(18)
	Vascular smooth muscle cells and macrophages	Increases chemotaxis of inflammatory cells, thereby amplifying vascular inflammation and triggering the formation of smooth muscle foam cells.	(6)
	Enterocyte	Regulates the migration of medullary immune cells to mucous membranes and plays an anti-inflammatory role	(7)
	Bone marrow endothelial cells	Activates the Y1R in macrophages to promote neural protection.	(19)
	Thymic epithelium	Protects thymus cell development	(5)
Monocyte system	Langerhans cells	Helps protect the skin against invading microbes	(20)
	Human monocyte-derived DC	Is involved in the maturation of dendritic cells	(21)
	Mouse bone marrow-derived monocytes and human peripheral blood monocytes	Is involved in the regulation of monocyte function	(22, 23)
	Mouse DC and macrophages	Performs anti-inflammatory role	(24)
	Airway macrophages	Is involved in the regulation of cytokine production and cellular activity of immune cells in asthma	(25)
	Retinal microglia	Is involved in the regulation of eye inflammation	(26)
	Primary hippocampal microglia	May be related to the immune response against sepsis	(27)
	N9 microglial cell line	Inhibits NO synthesis and IL-1β release	(28)
Lymphocyte	T and B lymphocytes	Is involved in lymphocyte autoregulation	(29, 30)
Granulocyte	Mastocyte	Executes a role in the infection and elimination of hepatitis virus	(30)

DC, dendritic cell; IL-1β, interleukin-1β; IL-6, interleukin-6; NO, nitric oxide.

The removal of the sympathetic nerve did not reduce the content of NPY in the spleen. Moreover, denervation retained high NPY expression in leukocytes isolated from renal grafts, implying that immune cells independently provide NPY (33). However, the NPY of immune cells is inducibly expressed rather than constitutively expressed. It appears that immune cells synthesize NPY only when stimulated or differentiated into mature cells (21, 22, 28, 29), and subsequently participate in the regulation of cytokine production and activity of immune cells (25). In addition, nerve growth factor promotes NPY synthesis by unstimulated T cells; however, this effect is not observed in B cells (29).

### NPYRs in the Immune System

NPYRs are widely expressed in immune cells, especially Y1R, which exists in almost every type of immune cell (**Table 2**). Y1R was initially detected in rat splenic lymphocytes. Although it shares 100% homology with Y1R receptors in the brain, its basal expression level is significantly lower than that measured in the frontal cortex (37). However, in a variety of immune cells, when functional activity is required, the basal expression of NPYRs is robustly upregulated after antigen stimulation or inflammatory stimulation (27, 36, 41). Moreover, NPY also plays a role in regulating the expression of its receptors (28).

**Table 2:** Neuropeptide Y receptors in immune cells.

Species	Tissue	Cell type	Receptorisoform	Molecular level	Reference
Rat	Peripheral blood	Granulocyte	Y1R, Y2R, Y5R	Protein	(34)
	Peripheral blood	Monocyte	Y1R, Y2R, but not Y5R	mRNA	(35, 36)
	Spleen	Lymphocyte	Y1R	mRNA	(37)
	Dental pulp	CD^43+^-granulocyte CD^4+^-lymphocyte	Y1R	Protein	(38)
	Air-pouch	Granulocyte	Y1R, Y2R, Y5R	Protein	(39)
	Retinal	CD11b^+^ microglia	Y1R, Y2R	Protein	(40)
Mouse	Bone marrow	Macrophage	Y1R, Y2R	mRNA	(6, 19)
	Bone marrow	Dendritic cell	Y1R, Y2R, Y4R, Y5R	mRNA	(24, 41)
	Lymph node	T/B lymphocyte	Y1R	mRNA	(42)
	Spleen	Leukocyte	Y1R, Y2R, Y4R, Y5R, Y6R	mRNA	(15)
	Adipose tissue	Macrophage	Y1R, Y2R	mRNA	(24)
	–	N9 Microglia cell line	Y1R, Y2R, Y5R	mRNA Y1 (mRNA and protein)	(28)
Human	Peripheral blood	Neutrophil	Y1R, Y2R, Y4R, Y5R	mRNA	(41)
	Infantile hemangioma	T/B lymphocyte (CD^45+^) and mast cells (tryptase)	Y1R	Protein	(43)
Pig	Hippocampi	Microglial	Y1R	mRNA	(27)

The expression of NPYRs is also altered depending on age and pathological status. The percentage of granulocytes in air pouch expressing Y1R, Y2R, and Y5R in adult rats was significantly higher than that noted in young and old rats; however, there was no difference between young and old rats (39). Moreover, Y4R, Y5R, and Y6R of tyrosine hydroxylase positive (TH+) cells were significantly increased in diabetic mice (15), and Y1R expression was also increased in the bone marrow-derived dendritic cells (BMDCs) of obese mice (24). Although homologous immune cells express various receptors, the expression of different Y receptor subtypes on a single cell and their effects on cell function warrant further investigation.

## NPY Directly Regulates Immune Cells

**Figure 2:**
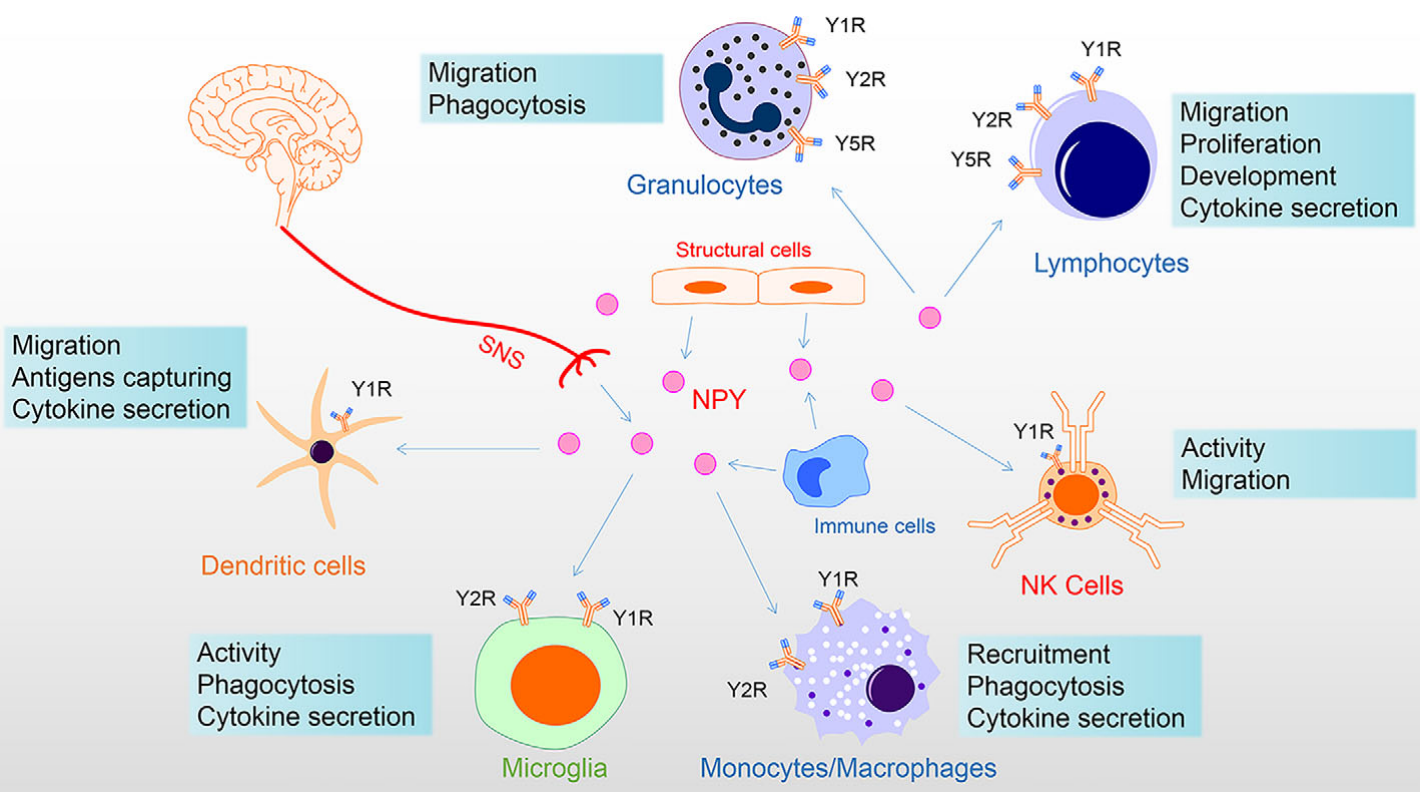
NPY directly regulates immune cells. NPY of the immune system derived from the secretion of the sympathetic nervous system, tissue structure cells, and immune cells. NPY plays multiple roles in immune cells, including via Y1R inhibiting activation and regulation of proliferation, differentiation, and cytokine secretion; via Y1R/Y2R/Y5R mediating phagocytosis and migration. Among these, Y1R has a bimodal effect on the immune system, showing both anti-inflammatory properties and specific pro-inflammatory effects. In addition, the Y1R mainly mediates the promotion of NPY, while the Y2/Y5 receptor mediates the inhibition of NPY. Therefore, the interaction of Y1R and Y2/Y5R is involved in the regulation of immune cells. In this figure, NPY receptor symbols on different immune cells symbolize their involvement in the diverse functional regulation of NPY. NK, natural killer; NPY, neuropeptide Y; SNS, sympathetic nervous system.

The innervation of immune organs constitutes the anatomical link between the nervous system and immune system. The sympathetic nerve fibers end in the parenchyma in close contact with immune cells (44). At this neuroimmune junction, the neurotransmitters released by sympathetic nerve endings can stimulate specific receptors on immune cells and affect the function of immune cells. For example, activation of the central amygdala (CeA) and paraventricular nucleus (PVN) corticotropin-releasing-hormone neurons control humoral immune responses in a T-cell-dependent manner by promoting splenic sympathetic output and releasing NE (45). Moreover, under the stimulation of inflammation, the content of NPY secreted by neurogenic, structural, and immune cells is elevated, regulating immune cell function in a paracrine or autocrine manner (46–48). Thus, endogenous NPY may serve as an immunoregulatory factor directly on NPYRs of immune cells (**Figure 2**).

### Lymphocytes

NPY-IR nerve fibers are in close contact with lymphocytes (46), and NPY produced by splenic sympathetic nerve endings facilitates nerve communication with TH+ leukocytes (15). It betokens that NPY is engaged in lymphocyte recruitment. Indeed, NPY preferentially mobilized CD4+ T cells at high doses, whereas it recruited IgM_low_CD_5+_CD_11b+_B cell subsets dose-dependently (49).

Additionally, the physiological concentration of NPY induces high-level adhesion of human and murine T cells to fibronectin in the resting-state by inciting β1 integrins. In humans, this may be due to Y2R activation providing a pre-adhesion signal for T cells (50). However, in rats, Y1R and Y5R also participate in lymphocyte mobilization, in which Y-5R activation is enhanced, and Y-1R tension inhibits adrenaline-induced leukocyte mobilization (35).

Discrepantly, NPY heightened the chemotaxis of lymphocytes in axillary and peripheral lymph nodes of mice, yet it restricted the chemotaxis of thymic lymphocytes and had no impact on splenic lymphocytes (51). Withal, the regulatory effect of NPY on chemotaxis is inhibited or absent in aged animals. Therefore, NPY may alter lymphocyte recruitment by modifying lymphocyte adhesion and tropism, depending on the activated receptor, age, and lymphocyte subsets.

NPY (10^−12^–10^−8^ M) inhibited lymphocyte proliferation *in vitro* (33). NPY significantly restrained lymphocyte proliferation in response to stimulation with mitogen Concanavalin A or lipopolysaccharide (LPS) (5). Moreover, this regulatory effect of NPY may be exerted by diminishing the production of IL-2; however, those effects are abrogated with aging (5). Furthermore, the Y1R pathway is involved in the supervision of B cell development in bone marrow, decreasing the number of pro-B, pre-B, and immature B cells and increasing that of mature B cells (52).

Interestingly, in humans, NPY also appears to preserve T and B lymphocytes from apoptosis, and promotes the proliferation of lymphocytes. In critically ill patients, the level of NPY is positively correlated with the total number of lymphocytes, T helper (Th) cells, and toxic T cells (53). Furthermore, serum NPY is negatively correlated with the expression of apoptosis-related molecule Fas/Fas ligand in Th cells and cytotoxic T cells, and negatively correlated with the expression of Fas in B cells (54). Early studies demonstrated that NPY (10^−12^–10^−6^ M) facilitates the proliferation of human colonic lamina propria lymphocytes by promoting the production of IL-1β in monocytes (55). Nevertheless, whether NPY has a positive role in lymphocyte survival and proliferation merits further research.

NPY also improves the Th2 inflammatory response in asthmatic patients (56). Indeed, NPY (10^−9^ M) upregulates the expression of IL-6 and IL-10 by human immature dendritic cells, promoting the secretion of IL-4 *via* Th2 polarization, and inhibits the production of interferon-gamma (IFN-γ) (57). It has been demonstrated at the animal level that activation of Y1R may be required for this process, as specific Y1R agonists have a significant inhibitory effect on Th1 polarization and promote autoimmune T cells to Th2 bias (42). However, Y1R holds a bimodal role in the immune system, serving as a potent negative regulator of T cells and a key activator of antigen-presenting function (58). While Y1R signaling can inhibit the activation of T cells, mice without a Y1R signal are resistant to Th1 cell-mediated inflammatory response, showing a decrease in the level of Th1 cell-promoting factor IL-12 and the production of IFN -γ. It may be that NPY stimulates Th1 cells to secrete Th1 (IL-2 and IFN -γ) and Th2 (IL-4) cytokines and directly stimulates Th2 cells to secrete IFN -γ (50).

### Natural Killer Cells

The NK cell is a critical immune cell in the body, playing an essential role in anti-tumor, anti-virus infection, and immune regulation (59). In the context of inflammation, NPY inhibits NK cell activity. NK cells need to be fully activated to exert their immune effects, and this process can be interrupted by Y1R signaling. Studies have demonstrated that human NK cell activity is negatively correlated with plasma NPY levels (60). Intravenous injection of NPY begets a dose-dependent inhibitory effect on splenic NK activity in rats (61). Moreover, the Y1R antagonist eliminates the inhibitory effect on NK cell activity (61).

The regulation of NPY on NK cells is characterized by heterogeneity. Analogously, large doses of NPY (50 μg/kg) intravenously mobilized activated NK cells, while low doses of NPY (0.1 μg/kg) significantly reduced the number of NK cells in the blood (49). Further, 15 min following the intraventricular injection of NPY (10^−9^ M), the number of NK cells in the blood increased, while the toxicity of NK cells decreased. However, 1 h and 24 h after the initial immunosuppression, NPY enhanced the activity of NK cells (62). Finally, NPY stimulated the NK activity in axillary lymph nodes and thymus of adult (24±2 weeks) and mature (50±2 weeks) animals, whereas it inhibited the NK activity in the spleen of young mice (12±2 weeks). Moreover, it decreased the cAMP level in the leukocytes of adult mice, indicating that the messenger may be involved in the regulation of NK cells by NPY (63).

### Dendritic Cells

In mice stimulated by inflammation, the maturation of DC and the production of inflammatory cytokines are hindered by NPY (24). Following stimulation, endogenous tonic activation of Y1R, Y2R, and Y5R inhibited the expression of pro-inflammatory factors. This tonic activation also inhibited the expression of maturation markers of DC. Indeed, NPY (10^−9^ M) failed to induce the phenotypic maturation of BMDCs. Nevertheless, its dose-dependent (10^−12^–10^−7^ M) effect caused transendothelial migration of immature DCs by binding to Y1R, and activating the extracellular signal-regulated kinase (ERK) and p38 mitogen-activated protein kinase. Furthermore, NPY plays an anti-inflammatory role by promoting the production of IL-6 and IL-10 in DCs, inducing T-cell to polarize toward Th2 (24).

However, the pro-migration effectiveness of NPY on DCs appears to play a specific pro-inflammatory role, leading to the aggravation of local inflammation in mice (64). NPY inhibited the expression of costimulatory molecules CD80 and CD86 in antigen-presenting cells (65). Similarly, BMDCs in Y1R-deficient mice exhibited an impaired phagocytic capacity for fluorescent-labeled ovalbumin and reduced their production of IL-12, thus failing to provide optimal stimulation to T cells (58). Interestingly, both human BMDCs and murine Langerhans cells can synthesize NPY (20, 21); hence, NPY may control the function of antigen-presenting cells in an autocrine-dependent manner to induce adequate adaptive immune responses.

### Granulocytes

The role of NPY in granulocytes is heterogeneous. It is based on both the dose of NPY, the diverse receptor activation, and the source of granulocytes. In the bronchoalveolar lavage fluid of the mouse asthma model, the concentration of NPY was positively correlated with the total count of leukocytes and eosinophils (66). Furthermore, NPY enhanced human neutrophil phagocytosis of *Escherichia coli* at low doses but did not alter respiratory burst. At high concentrations, NPY inhibited phagocytosis but enhanced respiratory burst (41).

However, various isoforms of NPYRs activation also play different roles. Activation of Y1R heightens granulocyte adhesion and phagocytosis of zymosan by rat granulocytes (34). On the contrary, activation of Y5R does not regulate its phagocytosis, but inhibits its adhesion (34). Indeed, Y1R antagonists possess an inhibitory potency on mice eosinophils (64). Moreover, Y2R of granulocytes appears to have an unusual opposite effect versus Y1/Y5R. Activation of Y1/Y5R inhibited the phagocytosis of zymosan by rat granulocytes (39), but potentiated phagocytosis by stimulating the production of reactive oxygen species in human neutrophils (41). In contrast, Y2/Y5R stimulation did not affect the phagocytosis of zymosan by rat granulocytes (39), but resulted in a substantial reduction in its production of reactive oxygen species (67).

NPY also has various impacts on granulocytes at different localities (68). NPY inhibits the function of blood granulocytes by activating Y1R. On the contrary, NPY and Y1R antagonist BIBP3304+NPY initiate the function of splenic granulocytes.

Regulation of granulocyte phagocytosis by NPY is manifested by different effects on heterologous particles. NPY holds the ability to enhance opsonin-dependent phagocytosis by human neutrophils, but has no or a slight inhibitory effect on opsonin-independent phagocytosis (69). It is comprehended that NPY is highly dependent on the local microenvironment and participates in the regulation of inflammation-related granulocyte function through the interaction of Y1, Y2, and Y5R.

### Monocytes/Macrophages

Under various pathological conditions, NPY has been identified as a chemical attractant and adjusts cell adhesion to regulate the recruitment of monocytes and macrophages in rodents (6, 18, 34). Endogenous NPY affects the recruitment of monocytes/macrophages by activating Y1R to decrease their adhesion and promote migration (6, 34). Besides, exogenous NPY possesses significant chemotactic properties for monocytes at physiological concentrations (10^−8^ –10^−10^ M) (70). However, NPY (10^−9^–10^−10^ M) inhibited the migration of macrophages (RAW264.7) to *Leishmania* (71). Moreover, NPY inhibited the recruitment of retroviral-infected monocytes to CNS. This process is linked to the upregulation of monocyte Y2R (72). Indeed, NPY enhances the adhesion of monocytes through Y2R (36). This effect may be due to diversity in the dominant receptor subtypes activated by NPY because Y1R mediates the promotion of NPY, whereas Y2/Y5R mediates the inhibition of NPY. These opposite effects principally depend on the activity of dipeptidyl peptidase 4 (34). Dipeptidyl peptidase 4 is an enzyme that terminates the activity of NPY on the Y1 receptor subtype, and its activity changes with age (73).

Hence, the effect of NPY on macrophage adhesion also shows time and age specificity (74). NPY [(10^−13^–10^−7^ M) with 10 min] significantly enhanced the adhesion ability of macrophages in mature (50±2 weeks) and aged (70±2 weeks) mice. At the same time, NPY did not affect the adhesion ability of macrophages in young (12±2 weeks) and adult (24±2 weeks) animals. However, NPY [(10^−11^ M) with 20–30 min] enhanced the adhesion ability of macrophages of mice at all ages, except aged mice. Furthermore, NPY (10^−13^–10^−8^ M) significantly stimulated the chemotactic capacity of macrophages in adult mice and, conversely, the NPY inhibitory chemotactic capacity of macrophages at other ages.

As discussed for the granulocyte, different NPYRs are activated, and the diversity in heterologous particles leads to complex effects of NPY on the phagocytic function of monocytes/macrophages. NPY (10^−10^ M) and LP-NPY (Y1/Y5R agonist, 10^−10^ M) significantly increased the phagocytosis of zymosan in monocytes, whereas NPY3-36 (Y2/Y5R agonist, 10^−10^ M) decreased the phagocytic capacity of monocytes (34). Nevertheless, Y5R agonist [(Ala31-Aib32) NPY] could not regulate the phagocytosis of zymosan by monocytes, inferring the role of Y1R in NPY-induced intensification of phagocytic function. However, BIBO3304 (Y1R antagonist, 10^−8^ M) and L152804 (Y5R antagonist, 10^−8^ M) both antagonized the NPY-induced increase in the phagocytic capacity of monocytes (34).

Furthermore, treatment with NPY (10^−12^–10^−6^ M) *in vitro* dose-dependently inhibited macrophage phagocytosis; yet, NPY (10^−14^–10^−6^ M) enhanced the release of H_2_O_2_ to kill foreign particles (75). NPY enhanced the production of peritoneal macrophage peroxide by simultaneously activating Y1R and Y2R (73). NPY (10^−12^–10^−8^ M) and PYY (10^−12^–10^−6^ M) boosted the oxidative burst involving the activation of protein kinase C in macrophages stimulated by phorbol myristate through Y1R and Y2R mediation. However, only Y2R signaling diminished the oxidative burst in zymosan-stimulated cells, compromising the signaling pathways after binding of zymosan to macrophage complement receptor 3 (76). This diverse impact mediated by receptor subtypes has led to many seemingly contradictory conclusions, and the effects of NPY on macrophage phagocytic function for different heterologous particles are also divergent.

NPY (10^−12^–10^−8^ M) significantly intensifies the phagocytosis of latex particles by mouse macrophages (74), while NPY (10^−10^–10^−5^ M) inhibited the phagocytic and killing ability of macrophages (RAW264.7) against *Leishmania* (77). Besides, co-treatment of RAW264.7 macrophages with melanocyte-stimulating hormone/NPY affects lysosomal activity by inhibiting the expression of lysosomal associated membrane protein 1. This hinders the maturation of phagosomes, but does not affect their antigen presentation function (4). When treated with NPY only, the phagocytic ability of macrophages to unopsonized *Escherichia coli* and *Staphylococcus aureus* biologicals was significantly reduced. In contrast, the phagocytic ability of antibody-modified biologicals and Fc receptor-mediated phagocytosis of Gram-positive biologicals were not significantly impaired. Moreover, the inhibitory effect of neuropeptides on phagocytic activity does not occur through downregulation of CD206 or macrophage receptor with collagenous structure expression. These two primary scavenging receptors in activated macrophages inhibit the activation of phagocytic pathways and the production of Fc receptor-related active oxidative products (78). Thus, the effect of NPY on mononuclear/macrophage phagocytosis is highly dependent on the type of foreign particles, peptide truncation, and the interaction of specific Y receptors.

A critical role of NPY is to modulate the secretion of cytokines by macrophages. Knockdown of NPY reduced the secretion of pro-inflammatory factors from monocytes/macrophages in animals (18, 64), revealing the pro-inflammatory effect of NPY on macrophages. Under stimulation by inflammation, NPY significantly increases the expression of tumor necrosis factor-alpha (TNF-α), C-reactive protein, and monocyte chemoattractant protein 1 (MCP1) in RAW264.7 macrophages by activating Y1R (79). Moreover, NPY (10^−9^ M) upregulated the expression of HMGB1, which is a potent inflammatory stimulus, in the absence of an inflammatory stimulus, and the protein kinase C/ERK pathway was required in this process (80).

NPY also has obvious anti-inflammatory effects. Inhibition of the Y1R signal-induced production of IL-12 and Y1R-knockout macrophages also provoke their response to inflammatory stimuli (M1 phenotype) without affecting their alternating activation phenotype (M2 phenotype) (81, 82). Consistently, NPY produced by adipose tissue macrophages inhibits the expression of pro-inflammatory genes IL-6, TNF-α, and nitric oxide synthase 2 (NOS2) and decreases the secretion of IL-6 and TNF-α through the autocrine and paracrine systems, thereby inhibiting the activity of M1-like adipose tissue macrophages (24). NPY transformed macrophages to the M2-like phenotype, while NPY (10^−6^ M) mainly stimulated the release of macrophage anti-inflammatory cytokines IL-10 and IL-1RA and prevented the release of pro-inflammatory cytokines (e.g., TNF-α, IL-12, and IL-6) (83). Furthermore, NPY activates Y1R in macrophages to protect sympathetic fibers and bone marrow cells through TGF-β secreted by the PI3K/AKT/mTOR/eIL4E signaling pathway (19). However, it is noteworthy that the anti-inflammatory properties of NPY are also age-specific, and NPY (10^−10^ M) has an inhibitory effect on the release of TNF-α and IL-2 in adult mice, but not in aged mice (74).

### Microglia

NPY similarly inhibits microglial activation, phagocytosis, and cytokine secretion. It has been demonstrated that intracerebral injection of NPY (10^−2^ g/L) can diminish microglial activation induced by 6-hydroxydopamine in rodents, and NPY inhibits microglial activation by binding to the translocator protein (TSPO) ligand [3H]PK11195 (84). Although NPY repressed microglial activity, it did not impair microglial survival. Instead, NPY (10^−6^ M) inhibited the activation of microglia induced by methamphetamine and shielded them from methamphetamine-induced death (85).

NPY decreases the secretion of pro-inflammatory factors by microglia. Specifically, exogenous NPY (0.01, 0.10, 0.50, and 1.0 × 10^−6^ M) dose-dependently inhibited TNF-α production in microglia induced by toll-like receptor 2 (TLR2) agonists (27). Furthermore, NPY (10^−6^ M) lessened the LPS-induced transcription and secretion of IL-1β and TNF-α by microglia; however, this consequence was prevented after inhibition of Y1R receptors (86). Thus, activation of Y1R reduces the production of pro-inflammatory factors in microglia. Indeed, following inflammatory stimulation, microglia release IL-1β, which promotes nitric oxide (NO) production through a nuclear factor-κB-dependent pathway, whereas NPY (10^−6^ M) inhibits the release of IL-1β, nuclear translocation of nuclear factor-κB and inducible NOS expression through Y1R activation, thereby reducing the production of NO (28).

Moreover, Y2R also exhibits specific anti-inflammatory properties. In the medial prefrontal cortex, Y2R agonists PYY_3–36_ reversed LPS-induced increases in NLR family pyrin domain containing 3 (NLRP3), caspase-1, adaptor apoptosis-associated speck-like protein containing a CARD (ASC), IL-1β levels. NPY further reversed LPS-induced increases in caspase-1 and ASC levels, while the Y2R antagonist (BIIE0246) blocked the effect of NPY. Additionally, the results of the enzyme-linked immunosorbent assay showed that NPY and PYY_3–36_ reversed the LPS-induced upregulation of IL-1β levels; this effect was also prevented by BIIE0246. These data suggest that NPY inhibits the NLRP3 pathway through Y2R to prevent neuroinflammation (87). In conclusion, the activation of Y1R and Y2R holds a particular inhibitory capacity in the secretion of pro-inflammatory factors.

The role of NPY on microglia is also revealed in its direction of migration and phagocytosis. NPY (10^−6^ M) significantly restrains IL-1β-induced microglial motility by inhibiting p38 activation and actin recombination through Y1R activation (88). Moreover, in an inflammatory environment, microglia are strongly activated through a process involving downstream phosphorylation of p38 mitogen-activated protein kinase (MAPK) and heat shock protein 27 (HSP27) and magnify their phagocytic capacity. Notably, NPY (10^−6^ M) significantly represses the above process through activation of Y1R, inhibiting LPS-induced Fc receptor-mediated phagocytosis (89).

Considering that NPY is more broadly expressed in the CNS and microglia act as specific immune cells in the CNS, their interaction may be more intimate. For instance, the density of NPY fibers in the arcuate nucleus (ARC) and PVN increased after microglial ablation, although NPY expression in the hypothalamus was unaltered (90). Activated microglia remain in close contact with NPY neurons and inhibit the expression of NPY (91). Furthermore, activation of microglial TLR4 in ARC results in restraint of Agouti-related protein (AgRP)/NPY activity (92), and the mechanism involved in this process may be mediated by inducible NOS-NO signaling (93).

Interestingly, caloric restriction-induced changes in NPY are not directly involved in inhibiting LPS-induced microglial activation, but may indirectly affect microglial activation through BT regulation (94). It was speculated that NPY directly regulates immune cells by triggering NPYRs, and indirectly affects them by regulating other physiological and pathological processes.

## NPY Indirectly Regulates the Immune Response

**Figure 3:**
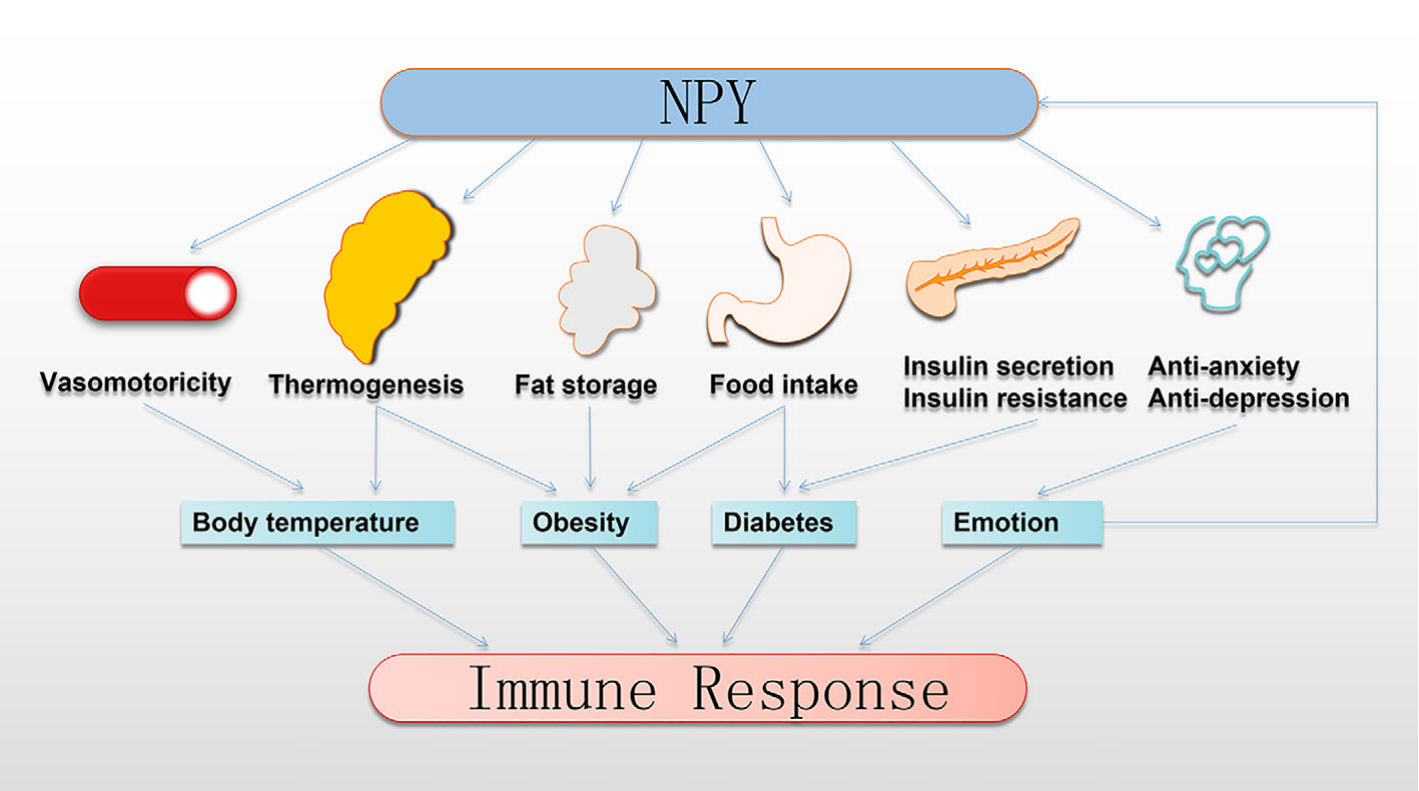
NPY indirectly regulates the immune response. NPY regulates thermoregulation, obesity, and development of diabetes by controlling the vasomotor of the skin, thermogenesis of BAT, fat storage in WAT, food intake, insulin secretion, and insulin resistance. Moreover, NPY plays anti-anxiety and anti-depression roles to transform emotional expression. Interestingly, this regulation by NPY profoundly impacts the immune response. BAT, brown adipose tissue; NPY, neuropeptide Y; WAT, white adipose tissue.

The immune system is susceptible to various physiological and pathological changes, including changes in BT, the occurrence of obesity, abnormal blood glucose, the development of depression and anxiety, and other immunomodulatory factors (95–98). Studies have confirmed that these immunomodulatory factors have a profound connection with NPY (**Figure 3**).

### NPY and BT

It is established that BT is an essential regulator of immune function. Hyperthermia stimulates the immune response, prompting primitive immune cells to enter the supporting environment of the lymph nodes; hypothermia reduces inflammation and immune response, affecting the circulation pattern of immune cells or the expression of transport molecules, mainly exerting an anti-inflammatory effect (95). Although there is no evidence confirming that NPY influences the immune response by regulating BT, some studies imply that NPY may indirectly affect the activation of immune cells through changes in BT (94). Indeed, NPY can influence the changes in BT by altering the vasomotor function of skin and the thermogenesis of brown adipose tissue (BAT), regulating the heat dissipation and heat production balance.

Investigations have shown that before the local temperature increases, the application of Y1R antagonists weakens the vasodilator response. However, after the local temperature increases, the Y1R antagonist has no impact on the response of vasodilators (99). In short, NPY is required to initiate vasodilation caused by local skin warming, but it does not participate in the maintenance of vasodilation. This performance may be due to the impact of NPY on the corresponding receptors of the skin sensory nerves. In contrast, the Y1R antagonism reduces the skin vasoconstriction response induced by systemic cooling. Furthermore, blocking the adrenergic α and β receptors does not entirely prevent the systemic cooling reflex response; however, this is achieved by the simultaneous application of NPYR antagonists (100). Thus, NE and NPY co-released by sympathetic nerves are involved in the contraction of skin blood vessels caused by cold stimulation to reduce heat dissipation. Collectively, the evidence reveals that the regulation of skin vasomotor function by NPY is highly dependent on environmental settings.

Thermogenesis is also an essential approach to BT regulation. A large body of evidence has supported that the hypothalamus is a vital transit station involved in BT regulation, altering BT by inducing the thermogenesis of BAT through the sympathetic nervous system (101). Intriguingly, NPY is widely expressed in the hypothalamus and has the highest concentration in the ARC. Furthermore, NPY neurons in ARC project to other hypothalamic regions closely related to heat production and appetite control, including the PVN and dorsomedial hypothalamus (DMH) (102). Chao et al. disclosed that knockdown of NPY in rat DMH led to an increase in the expression of uncoupling protein 1 (UCP1) in BAT and enhanced browning of inguinal white adipocytes tissue, thereby enhancing the thermogenic activity of classical BAT, which in turn led to an increase in BT (103). Further studies on the sympathetic denervation of inguinal white adipocytes tissue found that DMH NPY knockdown increased sympathetic outflow (103). Besides, DMH NPY overexpression led to a decrease in UCP1 expression in BAT (103). However, a thoroughgoing DMH NPY signaling pathway regulating BAT thermogenesis remains to be determined.

Consistent with these findings, our previous research reported that ARC NPY neurons projected onto PVN, inhibiting the sympathetic drive of BAT (8). More specifically, ARC NPY directly acting on the Y1 receptor on TH+ neurons in PVN reduces the expression of TH in PVN, which subsequently leads to a decrease in the expression of TH in the brainstem locus coeruleus, A1/C1 neurons, and nucleus tractus solitarii (8). Eventually, the activity of A1/C1 catecholaminergic neurons in the ventrolateral medulla associated with sympathetic ganglia is reduced, resulting in reduced sympathetic nerve outflow to peripheral tissues (e.g., BAT) (8). A study showed that the neuropeptide FF receptor 2 (NPFFR2) signal directly acts on ARC NPY neurons (104). Lack of this NPFFR2-induced tone on NPY neurons reduces the capacity of these neurons to control TH+ neurons in the PVN, with a consequent reduction in BAT function. Hence, the NPFFR2 signaling pathway in NPY neurons appears to affect the regulation of thermogenesis by controlling the expression level of NPY.

Additionally, ARC NPY activates the gamma-aminobutyric acid (GABA)-ergic neurons of the intermediate and parvicellular reticular nuclei through the nucleus tractus solitarii, thereby inhibiting the sympathetic premotor neurons, which ultimately leads to the inhibition of the thermogenic effect on BAT (105). The reduction of hypothalamic NPY after cold stimulation in wild-type rats is coupled with the increased expression of UCP1 in BAT, leading to heat generation (106). Paradoxically, when the third ventricle was injected with NPY (15 μg/μl in 2 h), the BT of the animal was rapidly increased. Although the mechanism of centrally administered NPY involved in inducing an increase in BT is unclear, increased exercise activity and increased peripheral vasoconstriction may be the cause of this phenomenon (107). Moreover, overexpression of NPY by NE neurons increases the BT of the animal after exposure to cold (108).

Overall, these findings strongly suggest that both hypothalamic ARC and DMH-derived NPY can directly control thermogenesis, at least in part by regulating sympathetic nerve output and BAT heat production. Besides, NPY derived from the sympathetic nervous system regulates heat dissipation by modifying the vasomotor of the skin and finally plays a role in regulating BT. However, the final result of BT regulation by NPY may be a combination of various effects.

### NPY and Obesity

Obesity produces a chronic inflammatory state, resulting in altered immune cell production, reduced T-cell variation, polarization of macrophages to a pro-inflammatory state, and an increase in multiple pro-inflammatory cytokines (96). Numerous immunomodulatory adipokines also affect the recruitment and polarization of immune cells, including leptin signals, adiponectin signals, cytokines, and chemokines secreted by adipose tissue (109). However, the occurrence of obesity involves a complicated synergy between extravagant food intake or reduction in energy expenditure, where the NPY system has been recognized as a critical player in the regulation of energy balance and pathophysiology of obesity.

As discussed earlier, NPY influences energy expenditure by regulating BAT thermogenesis and energy intake by regulation of appetite. Within the ARC of the hypothalamus, two significant groups of neurons play essential roles in the regulation of energy homeostasis: one group coexpresses NPY/AgRP and promotes food intake. In contrast, the other group coexpresses cocaine- and amphetamine-related transcript and proopiomelanocortin, decreasing appetite (110). Notably, NPY is one of the most potent appetite-promoting factors identified thus far. Its feeding stimulation is mediated through Y1R and Y5R in the hypothalamus and inhibition of ARC cocaine- and amphetamine-related transcript neurons (110). Reversely, Y2R appears to have an antagonistic effect on Y5R (111). In the hypothalamus of fasting and obese rats, NPY levels are elevated and correlate with food intake (13). Furthermore, acute exercise-induced feeding in mice requires activation of ARC NPY neurons (112).

NPY also plays a role in regulating feeding behavior in other nuclei. Firstly, DMH NPY gene silencing improves overeating and obesity induced by a high-fat diet (103); in turn, DMH NPY overexpression leads to increased food intake and weight gain, aggravating diet-induced eating disorders and obesity (113). Besides, the expression of DMH-NPY is inhibited by ARC GABAergic-proopiomelanocortin neurons (114), which may contribute to reasonable control of food intake. Secondly, NPY in the lateral hypothalamus increased rat food intake of free choice high-fat high-sucrose and chow diets and Y5R antagonists prevented all of the above results. In contrast, Y1R antagonists only played a significant role in chow-fed rats (115). Thirdly, selective activation of CeA NPY neurons leads to increased food intake and reduced energy expenditure (116). Importantly, selective lack of NPY in CeA neurons attenuates the obesity phenotype, whereas excessive production of NPY in CeA further enhances that phenotype (116).

Peripheral NPY also plays a crucial role in promoting adipocyte proliferation and differentiation and adipose storage in white adipose tissue. Adipose tissue-derived NPY promotes the proliferation and differentiation of mesenchymal stem cells and preadipocytes, a process thought to occur through Y1R, Y2R, and Y5R (117). Throughout differentiation, NPY mediates the increased expression of reactive oxygen species, peroxisome proliferator-activated receptor-γ, and CCAAT/enhancer-binding protein alpha. It also decreased the expression of UCP1, leading to increased lipid accumulation in terminal differentiation (118).

### NPY and Diabetes

In diabetes, the proliferation of T cells and macrophages is altered, and the functions of NK cells and B cells are impaired, manifesting as abnormalities of innate and adaptive immunity (97). The key to the development of diabetes is insufficient insulin secretion and insulin resistance, and NPY is involved in these processes. Researches have revealed that NPY plays a vital role in the regulation of blood glucose homeostasis in diabetic models (119). The ability of AgRP neurons to induce insulin resistance depends on the expression of NPY (120), and the application of NPY in the CNS has been shown to efficiently reduce sympathetic activation of BAT and improve systemic insulin sensitivity (8, 107). Therefore, the NPY-dependent regulation of systemic insulin sensitivity is consistent with reducing energy expenditure in mice under fasting conditions, possibly by regulating the sympathetic activation of BAT.

DMH NPY knockdown plays a therapeutic role in impaired glucose homeostasis in rats (121). Overexpression of NPY in DMH decreased the expression of UCP1 in BAT, leading to insulin resistance (122); however, the downregulation of DMH NPY improved glucose homeostasis and enhanced insulin sensitivity (103). Downregulation of DMH NPY improves hepatic insulin sensitivity in high-fat diet rats by activating the hepatic PI3K/AKT insulin signaling pathway (123). The central mechanism of this effect may be that DMH NPY projects to the dorsal motor nucleus of the vagus nerve and subsequently regulates glucose homeostasis through hepatic vagal efferents, as hepatic vagotomy eliminates the inhibitory effect of DMH NPY knockdown on hepatic glucose production (124).

Additionally, previous data persuasively suggested that the direct effect of NPY on insulin release from isolated islets is inhibitory, while the central effect of NPY indirectly leads to an increase in plasma insulin (125). The Y1R mediates the inhibitory impact of NPY on insulin secretion on islet β-cells (126). Given the effects of NPY on the central stimulation of feeding behavior in rats, it is logical that NPY induces delayed and transient increases in circulating insulin. Furthermore, NPY can rapidly and transiently induce the phosphorylation of ERK1/2 to promote β-cell proliferation (126, 127).

### NPY and Emotion

Increasing data indicate that our emotional and immune states have complicated and bidirectional relationships with each other. These observations paved the way for the new concept of “affective immunology” that has been proposed (98). Studies have confirmed that NPY neurons are involved in the regulation of anxiety in mice (128). Moreover, direct injection or overexpression of NPY in the brain exerts anti-anxiety effects in rodents (129), and knockout of NPY exacerbates the anxiety phenotype in mice (130). In rodents, the alleviation of anxiety caused by overexpression of Y1R also corroborates the above findings (131). Similarly, knockout of Y1R leads to anxiety outcomes (132), and the intranasal administration of Y1R agonists is sufficient to prevent anxiety (133). Moreover, specific Y5R agonists were injected into the PVN, resulting in a reduction in anxiety-related behaviors in animals (134). Accordingly, Y1R and Y5R have overlapping regulatory effects on anxiety (135). Conversely, activation of the Y2R is anxiogenic by inhibiting GABAergic input (136), and treatment with specific Y2R antagonists can reduce anxiety behavior in rodents (137). Surprisingly, overexpression of NPY in cells that regularly express NPY in mice did not lead to the expected reduction in anxiety-like behavior (138).

The level of plasma NPY is significantly increased in patients with depression (139), and the NPY gene can be used as a risk gene for severe depression (140). However, NPY expression in the brain decreases overall during depression (13). Consequently, intraventricular infusion of NPY or intranasal delivery of NPY to the brain effectively prevents depressive behavior in animal models (133, 141). LP-NPY (Y1R and Y5R agonists) is as useful as NPY and presents therapeutic potential in preventing the development of depressive-like behavior (142). Nevertheless, Y5R antagonists also play an antidepressant role through the MAPK/ERK and PI3K signaling pathways (143). Besides, Y2R plays an antidepressant role by inhibiting the NLRP3 signaling pathway in LPS-induced depression model rats (87). Intriguingly, in turn, the depression-induced increase in NPY expression affects immune cell recruitment and cytokine secretion (18).

Taken together, either the indirect regulation of NPY neurons in the brain or the direct promotion of NPY secreted by sympathetic ganglia and adipose tissue to peripheral organs is closely related to various regulatory factors of the immune response. This ultimately influences the immune response. Therefore, it is conceivable that NPY directly regulates the immune response through these immunomodulatory factors.

## Summary and Prospect

Existing data indicate that the regulatory effect of NPY on the immune response can be either through the NPYR that directly acts on the surface of immune cells or indirectly through the regulation of physiological or pathological conditions such as BT, obesity, glucose metabolism, and mood (**Figures 2**, **3**). In summary, NPY has a variety of regulatory effects on immune cell activity, including proliferation, differentiation, cytokine secretion, migration, and phagocytosis, some of which are even contradictory. Considering the difference in the expression of NPYRs in different states of cell differentiation and activation, the different biological activities of NPY in immune cell populations can be interpreted. The complicated role of NPY and its signaling can be better understood if we consider that the role of NPY and the complexity of its signal transduction in intricate physiological or pathological contexts are correlated to the need for various types of regulation in a wide range of immune cell types to maintain appropriate homeostasis. Furthermore, it is vital to consider that the impact of NPY on the immune function of the body should be based on the overall effect, not ignoring its indirect effects.

Prospectively, NPY analogs with higher human NPYR selectivity, and different functional properties, have been developed in recent years (144). As discussed in this article, NPYR subtypes are an interesting target in biomedical research and drug development due to their diverse physiological and pathophysiological roles. Biased NPYR agonists/antagonists can be used to specify a signaling pathway for a specific biological effect. For example, in the case of Y1R and Y2R signaling, they can demonstrate which receptor subtype dominates the anti-inflammatory effect of NPY, and further clarify the downstream complex signaling pathways that mediate this effect. Furthermore, short NPYR agonists with different properties can be used as “shuttles” to target immune cells, accurately guide covalently linked therapeutic drugs to reach the cell surface, and enter cells through human NPYR-mediated internalization transfer to achieve precise therapy. This observation will help find treatments that target specific pathways of human disease without affecting other signal-transduction pathways and lessen side effects. Future research in this field is warranted to produce fruitful results related to the development of unique immunotherapies.

## Author Contributions

W-cC, H-fH, and SL contributed to the conception and design of the review. W-cC drafted and finalized the manuscript. H-fH and SL contributed equally to writing the review. H-fH and SL revised the manuscript and provided critical advice on the content of the manuscript. All authors contributed to the article and approved the submitted version.

## Funding

This work was supported by the Quanzhou Science and Technology Project (2017Z014) and Natural Science Foundation of Fujian Province (2018J01289).

## Conflict of Interest

The authors declare that the research was conducted in the absence of any commercial or financial relationships that could be construed as a potential conflict of interest.

## Abbreviations

NPY, Neuropeptide Y; PYY, Peptide YY; CNS, Central nervous system; NE, Norepinephrine; NPYRs, Neuropeptide Y receptors; BT, Body temperature; TH+, Tyrosine hydroxylase-positive; BMDCs, Bone marrow-derived dendritic cells; CeA, Central amygdala; PVN, Paraventricular nucleus; LPS, Lipopolysaccharide; Th, T helper; IFN-γ, Interferon-gamma; NK, Natural killer; DCs, Dendritic cells; ERK, Extracellular signal-regulated kinase; TNF-α, Tumor necrosis factor-alpha; MCP1, Monocyte chemoattractant protein 1; NOS2, Nitric oxide synthase 2; TLR2, Toll-like receptor 2; NO, Nitric oxide; NLRP3, NLR family pyrin domain containing 3; ASC, Adaptor apoptosis-associated speck-like protein containing a CARD; MAPK, Mitogen-activated protein kinase; HSP27, Heat shock protein 27; ARC, Arcuate nucleus; AgRP, Agouti-related protein; BAT, Brown adipose tissue; DMH, Dorsomedial hypothalamus; UCP1, Uncoupling protein 1; NPFFR2, Neuropeptide FF receptor 2; GABA, Gamma-aminobutyric acid.
